# Derivation and application of a composite annoyance reaction construct based on multiple wind turbine features

**DOI:** 10.17269/s41997-018-0040-y

**Published:** 2018-04-11

**Authors:** David S. Michaud, Leonora Marro, James McNamee

**Affiliations:** 1 Consumer & Clinical Radiation Protection Bureau, Non-Ionizing Radiation Health Sciences Division, Environmental and Radiation Health Sciences Directorate, Health Canada, 775 Brookfield Road, Ottawa, ON K1A 1C1 Canada; 2 Environmental Health Science and Research Bureau, Population Studies Division, Biostatistics Section, Health Canada, Ottawa, ON K1A 0K9 Canada

**Keywords:** Noise, Principal component analysis, Renewable energy, Community surveys, Bruit, Analyse en composantes principales, Énergie renouvelable, Enquêtes communautaires

## Abstract

**Objectives:**

Noise emissions from wind turbines are one of multiple wind turbine features capable of generating annoyance that ranges in magnitude from *not at all annoyed* to *extremely annoyed*. No analysis to date can simultaneously reflect the change in all magnitudes of annoyance toward multiple wind turbine features. The primary objective in this study was to use principal component analysis (PCA) to provide a single construct for overall annoyance to wind turbines based on reactions to noise, blinking lights, shadow flicker, visual impacts, and vibrations evaluated as a function of proximity to wind turbines.

**Methods:**

The analysis was based on data originally collected as part of Health Canada’s cross-sectional Community Noise & Health Study (CNHS). One adult participant (18–79 years), randomly selected from dwellings in Ontario (ON) (*n* = 1011) and Prince Edward Island (PEI) (*n* = 227), completed an in-person questionnaire. Content relevant to the current analysis included the annoyance responses to wind turbines.

**Results:**

The first construct tested in the PCA explained 58–69% of the variability in total annoyance. Reduced distance to turbines was associated with elevated aggregate annoyance scores among ON and PEI participants. In the ON sample, aggregate annoyance was effectively absent in areas beyond 5 km (mean 0.12; 95% CI 0.00, 1.19), increasing significantly between (2 and 5] km (mean 2.13; 95% CI 0.92, 3.33), remaining elevated, but with no further increase until (0.550–1] km (mean 3.37; 95% CI 3.02, 3.72). At ≤ 0.550 km, the average overall annoyance was 3.36 (95% CI 2.03, 4.69). In PEI, aggregate annoyance was essentially absent beyond 1 km; i.e., (1–2] km (mean 0.21; 95% CI 0.00, 0.88); (2–5] km (mean 0.00; 95% CI 0.00, 1.37); > 5 km (mean 0.00; 95% CI 0.00, 1.58). Annoyance significantly increased in areas between (0.550 and 1] km (mean 1.59; 95% CI 1.02, 2.15) and was highest within 550 m (mean 4.25; 95% CI 3.34, 5.16).

**Conclusion:**

The advantages and disadvantages to an aggregated annoyance analysis, including how it should not yet be considered a substitute for relationships based on changes in high annoyance, are discussed.

**Electronic supplementary material:**

The online version of this article (10.17269/s41997-018-0040-y) contains supplementary material, which is available to authorized users.

## Résumé

**Objectifs:**

Le bruit est l’une des nombreuses caractéristiques des éoliennes susceptibles de causer de la gêne selon une fourchette allant de *pas du tout gênant* à *extrêmement gênant*. Aucune analyse n’a pu jusqu’à maintenant exprimer simultanément le changement de tous les niveaux de gêne en lien avec les nombreuses caractéristiques des éoliennes. Le principal objectif de cette étude était d’utiliser l’analyse en composantes principales (ACP) pour obtenir un indice unique de la gêne globale causée par les éoliennes (d’après les réactions au bruit, aux lumières clignotantes, l'effet stroboscopique, aux impacts visuels et aux vibrations), évalué en fonction de la proximité des éoliennes.

**Méthode:**

Cette analyse était fondée sur des données recueillies à l’origine dans le cadre de l’Étude transversale sur le bruit ambiant et la santé (ÉBAS) de Santé Canada. Des participants adultes (18 à 79 ans), un par ménage, sélectionnés au hasard en Ontario (*n* = 1011) et à l’Île-du-Prince-Édouard (*n* = 227) ont rempli un questionnaire en personne. Les données pertinentes pour cette analyse étaient les réactions de gêne à l’égard des éoliennes.

**Résultats:**

La première composante principale de l’ACP a expliqué de 58% à 69% de la variabilité de la gêne totale. Plus les éoliennes étaient proches, plus les indices de gêne globaux étaient élevés chez les participants de l’Ontario et de l’Île-du-Prince-Édouard. Dans l’échantillon de l’Ontario, la gêne globale était essentiellement absente dans les zones situées au-delà de 5 km (moyenne 0,12; IC à 95% 0,00, 1,19), augmentant significativement entre (2 et 5] km (moyenne 2,13; IC à 95% 0,92, 3,33), restant élevée mais sans augmentation supplémentaire jusqu’à (0,550–1] km (moyenne 3,37; IC à 95% 3,02, 3,72). À ≤ 0,550 km, la gêne globale moyenne était de 3,36 (IC à 95% 2,03, 4,69). À l’Île-du-Prince-Édouard, la gêne globale était essentiellement absente au-delà de 1 km; c’est-à-dire (1–2] km (moyenne 0,21; IC à 95% 0,00, 0,88); (2–5] km (moyenne 0,00; IC à 95% 0,00, 1,37); > 5 km (moyenne 0,00; IC à 95% 0,00 1,58). La gêne globale augmentait significativement dans les zones comprises entre (0,550 et 1] km (moyenne 1,59; IC à 95% 1,02, 2,15) et était la plus élevée à l’intérieur de 550 m (moyenne 4,25; IC à 95% 3,34, 5,16).

**Conclusion:**

Les avantages et les inconvénients d’une analyse de la gêne globale, y compris le fait qu’elle ne peut pas encore être considérée comme pouvant remplacer les relations fondées sur les changements des niveaux de gêne élevés, sont décrits.

## Introduction

Annoyance, in particular *high annoyance* with noise, remains one of the most studied reactions in socio-acoustic surveys with widespread agreement that the prevalence of annoyance increases with increasing noise levels. Largely in response to the formative publication (Schultz 1978), scientists have defined “highly annoyed,” in the absence of a directly named option, as a response to a social survey question on noise annoyance with a response in the top 27–29% on an anchored numerical scale, or in the top two categories on a 5-point adjectival scale. The relative importance assigned to lower magnitudes of annoyance was marginalized because of the poor association with activity interference and greater susceptibility to influence by non-acoustic variables. In contrast, high annoyance was considered to reflect an adverse reaction clearly directed toward the sound emitted by a source; a reaction less swayed by non-acoustic factors and more readily mitigated by noise ordinance (Schultz 1978). Efforts to quantify the change in the prevalence of high annoyance, or its equivalent, have since dominated the vast majority of community noise annoyance studies.

Assessing community response to changes in wind turbine noise (WTN) is a more recent area of investigation. The science base demonstrates a predictable growth in the percentage of a community highly annoyed by WTN with an increase in long-term A-weighted WTN levels (Kuwano et al. 2014; Michaud et al. 2016a; Mroczek et al. 2012; Pawlaczyk-Łuszczyńska et al. 2014; Pedersen and Persson Waye 2004, 2007; Pedersen et al. 2009) or reduced distance between dwellings and wind turbines (Shepherd et al. 2011). Michaud et al. (2016b) reported that annoyance toward wind turbines was not limited to noise emissions. This was perhaps most clearly demonstrated by the observation that in areas with the highest calculated WTN levels, the prevailing annoyance responses were not directed toward WTN, but rather toward blinking lights, shadow flickers, or the visual impacts in general.

Utility scale wind turbines are complex and their features can invoke a range of annoyance reactions from nearby communities. The objective of the following analysis was to develop an aggregate measure of annoyance, based on multiple wind turbine features, to assess the change in annoyance in relation to proximity to wind turbines. As such, aggregate annoyance could supplement traditional assessments, which are currently based primarily on annoyance toward WTN. Changes in aggregate annoyance can also be assessed in relation to multiple measures of health/well-being and therefore support stakeholder efforts to mitigate adverse impacts on nearby communities. The authors investigate this possibility in an accompanying publication (Michaud et al. 2018).

Ultimately, the approach offers a more comprehensive way of describing *overall* community annoyance toward multiple wind turbine features that could not otherwise be achieved by focusing only on *high* annoyance toward WTN, or any other feature in isolation.

## Methods

### Study characteristics

The current study is a secondary analysis of the data collected as part of Health Canada’s CNHS. Any duplication of the methods already presented is intentional and considered the minimum necessary for the current analysis to stand on its own. The study characteristics have been described elsewhere (Michaud et al. 2016b). Briefly, dwellings were identified from two Canadian provinces. The Ontario (ON) and Prince Edward Island (PEI) sampling regions included 315 and 84 wind turbines and 1011 and 227 dwellings, respectively. The wind turbine electrical power outputs ranged between 660 kW and 3 MW (average 2.0 ± 0.4 MW). To maximize sampling in areas where potential impacts from WTN exposure would be most likely to occur, a “take-all” sampling strategy was employed for all identified dwellings within approximately 600 m of a wind turbine. The remaining dwellings were selected randomly up to approximately 11 km. From each dwelling, one participant between the ages of 18 and 79 years was randomly chosen to participate. Participants were not compensated for their participation.

This study was approved by the Health Canada and Public Health Agency of Canada Review Ethics Board (Protocol #2012-0065 and #2012-0072).

### Calculated distance between dwellings and wind turbines and environmental measures

Locations of wind turbines and dwellings were estimated using global positioning system (GPS) data. Wind turbine sound power levels, sound pressure levels at dwellings and calculated shadow flicker levels at dwellings are provided elsewhere. Briefly, wind turbine sound power levels in the CNHS were assessed following the IEC 61400-11 standard to support the calculation of outdoor wind turbine sound pressure levels at dwellings based on the ISO 9613-2 sound propagation standard module in CadnaA (Keith et al. 2016a, b). Shadow flicker exposure at dwellings was calculated using the WindPro software, which estimates shadow flicker exposure from all possible visible wind turbines from a particular dwelling (Voicescu et al. 2016).

### Data collection

The full study questionnaire is available elsewhere (Michaud et al. 2016b). Statistics Canada trained interviewers conducted in-person home interviews between May 2013 and September 2013. In addition to basic demographic variables and previously validated content, the questionnaire’s *perception module* included several questions on annoyance to multiple wind turbine features. Participants were asked to indicate their magnitude of annoyance toward noise, blinking lights, shadows or flickers of light, visual impacts, and vibration or rattles noticed indoors that coincided with a participant’s recollection of wind turbine operations. Annoyance response categories included *not at all*, *slightly*, *moderately*, *very*, and *extremely*.

### Statistical methodology

#### Derivation of an aggregate annoyance construct

Cronbach’s alpha was calculated to determine the internal consistency of the 5 wind turbine features associated with annoyance (i.e., noise, blinking lights on the turbine nacelle, vibrations, visual impact, and shadow flicker). A principal component analysis (PCA) was then conducted to discover and summarize the pattern of intercorrelations among the 5 evaluated wind turbine features (i.e., “annoyance features”) in order to derive a single criterion variable for annoyance based on the 5 wind turbine features. A strength of PCA is that it does not assume equivalence between the various wind turbine features in how they load onto an overall measure of annoyance. Principal components having an eigenvalue greater than 1 indicate that they adequately account for the covariation among the annoyance variables, and therefore are further considered for analysis (Cattell 1966; Hatcher and Stepanski 1994). In order to derive an overall annoyance construct, which would reflect total annoyance toward multiple turbine features, numerical values needed to be assigned to the adjectival scale as follows: 0: not at all, 1: slightly annoyed, 2: moderately annoyed 3: very annoyed, 4: extremely annoyed. Participants were also assigned to the “not at all annoyed” category if they indicated (1) that wind turbines were not visible/audible indoors or outdoors from anywhere on their property and (2) they did not perceive vibrations or rattling indoors during wind turbine operations. The annoyance construct based on the PCA is a linear combination of the original annoyance variables and represents a parsimonious description of the dependence structure, which conveys approximately the same amount of information expressed by the original variables (albeit on a numerical scale as compared to an adjectival scale). PCA constructs are treated as continuous variables.

#### Relationship between distance and aggregate annoyance

An analysis of variance (ANOVA) was performed based on the constructs derived from PCA to compare overall annoyance levels across different distance categories between wind turbines and dwellings. Distance to the nearest turbine was chosen as the primary exposure variable to model aggregate annoyance. Distance and log distance were found to be highly correlated to calculated dBA (Pearson’s *r* values = − 0.93 and − 0.95, *p* < 0.0001, respectively) and dBC levels (Pearson’s *r* values = − 0.90 and − 0.85, *p* < 0.0001, respectively). To facilitate comparisons to existing studies, a parallel analysis based on calculated A- and C-weighted WTN levels is available as supplemental material.

The two provinces were analyzed separately for ease of interpretation of results. The assumptions of the ANOVA were verified using the Anderson-Darling test for normality and Levene’s test for equal variance of the residuals. When the assumptions were not satisfied, non-parametric methods were applied. Final distance to the nearest turbine categories were retained from the original analysis (Michaud et al. 2016b) as follows: {≤ 0.550; (0.550–1]; (1–2]; (2–5]; > 5 km}. The analysis was repeated for both A- and C-weighted WTN levels using the originally defined categories (Michaud et al. 2016a). Tukey pairwise comparisons were carried out when the overall significance of the ANOVA model was less than 0.05, to ensure that the overall type I (false-positive) error rate is less than 0.05. The contribution that each annoyance variable had on the overall annoyance construct was also investigated by independently removing each annoyance variable from the 5-factor PCA to yield a modified 4-factor annoyance construct outcome. The analysis was conducted with and without the 110 participants who reported to personally benefit from having wind turbines in the area in recognition that high annoyance toward WTN was essentially absent in these participants (Michaud et al. 2016b).

The data analysis for this paper was generated using the SAS/STAT software, Version 9.2 of the SAS System for Windows 7. Unless otherwise indicated, a 5% significance level (*α* = 0.05) was implemented throughout.

## Results

### Proximity to wind turbines, response rates, and sample characteristics

Of the 2004 potential dwellings, 1570 addresses were considered to be in-scope dwellings, from which 1238 occupants agreed to participate in the study (606 males, 632 females). This produced a final calculated response rate of 78.9%, which did not differ by proximity to wind turbines in either province (*p* = 0.9971). A characterization of the study demographics, reported health effects and out-of-scope locations has been provided elsewhere (Michaud et al. 2016b). Table 1 summarizes corresponding A- and C-weighted noise levels (dBA and dBC, respectively) and modeled shadow flicker in maximum minutes of exposure at the dwelling per day (SFm) for each distance group modeled in the study.

**Table 1:** Sample exposure characteristics

Sample characteristics	Calculated distance between dwelling and nearest wind turbine (km)					
	≤ 0.550	(0.550, 1]	(1, 2]	(2, 5]	> 5	Chi-square^a^ *p* value
ON						
dBA mean [min, max]	41.13 [37.40, 44.60]	38.43 [31.80, 43.60]	33.21 [26.30, 40.40]	27.36 [22.60, 30.90]	8.69 [0.00, 18.20]	
dBC mean [min, max]	58.35 [55.00, 63.00]	56.49 [52.00, 61.00]	53.58 [47.00, 58.00]	50.21 [47.00, 54.00]	32.41 [0.00, 45.00]	
SFm mean [min, max]	33.76 [0.00, 79.00]	15.73 [0.00, 68.00]	5.78 [0.00, 23.00]	0.00 [0.00, 0.00]	0.00 [0.00, 0.00]	
Response rate *n* (%)	34 (72.3)	488 (80.1)	396 (78.7)	42 (82.4)	51 (77.3)	0.7009
Personal benefits^b^ *n* (%)	15 (44.1)	55 (11.5)	16 (4.3)	1 (2.6)	0 (0.0)	< 0.0001
Visible^c^ *n* (%)	34 (100.0)	474 (97.1)	348 (88.3)	32 (78.0)	6 (11.8)	< 0.0001
Audible^d^ *n* (%)	26 (76.5)	325 (66.6)	111 (28.0)	5 (11.9)	1 (2.0)	< 0.0001
PEI						
dBA mean [min, max]	42.87 [39.40, 46.10]	38.95 [34.30, 43.20]	32.47 [29.10, 37.20]	22.26 [14.60, 29.90]	11.10 [0.00, 18.20]	
dBC mean [min, max]	60.92 [58.00, 63.00]	58.20 [55.00, 62.00]	53.19 [51.00, 57.00]	45.44 [36.00, 54.00]	32.08 [0.00, 43.00]	
SFm mean [min, max]	40.11 [0.00, 78.00]	18.08 [0.00, 47.00]	1.69 [0.00, 20.00]	0.00 [0.00, 0.00]	0.00 [0.00, 0.00]	
Response rate *n* (%)	37 (77.1)	95 (79.2)	67 (75.3)	16 (64.0)	12 (100.0)	0.1666
Personal benefits^b^ *n* (%)	8 (21.6)	6 (6.4)	5 (8.5)	3 (23.1)	1 (10.0)	0.0651
Visible^c^ *n* (%)	34 (94.4)	94 (98.9)	59 (88.1)	2 (12.5)	2 (16.7)	< 0.0001
Audible^d^ *n* (%)	30 (83.3)	73 (76.8)	15 (22.4)	0 (0.0)	0 (0.0)	< 0.0001

*dBA* calculated outdoor A-weighted wind turbine noise levels, *dBC* calculated outdoor C-weighted wind turbine noise levels, *SFm* calculated maximum shadow flicker at dwellings (min/day)

^a^Chi square test of independence, testing the independence between the sample characteristic and distance groups

^b^Participants reported to receive personal benefit through rent, payments, or other indirect benefits such as a hall or community centre for having wind turbines in their area

^c^Participants reported that wind turbines were visible from anywhere on their property when at home

^d^Participants reported that wind turbines were audible when inside or outside their home

## Results based on principal component analysis

### Derivation of aggregate annoyance construct

The analysis of self-reported high annoyance (assessed individually) toward several annoyance features in relation to WTN levels has been presented in a separate paper (Michaud et al. 2016b). Table 2 presents the Spearman correlation coefficient between the various self-reported annoyances. All correlation coefficients were significant with *p* < 0.0001.

**Table 2:** Spearman correlation coefficient for the 5 self-reported annoyances, *n* = 1226

	Visual	Blinking lights	Shadow flickers	Vibrations
Noise	0.56	0.51	0.52	0.31
Visual		0.64	0.49	0.24
Blinking lights			0.55	0.21
Shadow flickers				0.23

In all cases, the Spearman correlation coefficient was significant with *p* < 0.0001

Table 3 presents the summary results from the PCA as well as the ANOVA model relating the first PCA construct to distance. Results relating the first construct of PCA to both A- and C-weighted WTN levels are presented in the supplemental material. A total of 1226 participants responded to all 5 wind turbine annoyance features.

**Table 3:** Summary of aggregated annoyance, principal component analysis, and ANOVA models based on the first construct of PCA, when all 5 annoyance variables are included in the construct, as well as removing one annoyance variable at a time from the construct

	Variable removed from the overall aggregate annoyance construct						
	None (overall)	Personal benefits^a^	Vibration annoyance	Noise annoyance	Visual annoyance	Shadow flicker annoyance	Blinking lights annoyance
Cronbach’s alpha	0.82	0.82	0.85	0.76	0.75	0.76	0.75
Summary statistics based on addition of annoyance variables							
*N*	1226	1116	1233	1226	1226	1227	1226
Mean	2.25	2.36	2.20	1.75	1.56	1.83	1.69
Median	0.00	0.00	0.00	0.00	0.00	0.00	0.00
Std dev	3.89	3.98	3.75	3.14	2.90	3.13	2.97
Std error	0.11	0.12	0.11	0.09	0.08	0.09	0.08
Minimum^b^	0	0	0	0	0	0	0
Maximum^c^	20	20	16	16	16	16	16
Values from PCA							
Eigenvalue of construct 1^d^	2.91	2.91	2.77	2.35	2.32	2.36	2.31
Proportion of variance in total annoyance explained by construct 1	0.58	0.58	0.69	0.59	0.58	0.59	0.58
	PEI distance^e^ (km)						
*p* value of distance category^f^	< 0.0001	< 0.0001	< 0.0001	< 0.0001	< 0.0001	< 0.0001	< 0.0001
Pattern of differences between distance categories^g^	A, A, B, B, B	A, B, C, C, C	A, A, B, B, B	A, B, C, C, C	A, A, B, B, B	A, A, B, B, B	A, A, B, B, B
	ON distance^e^ (km)						
*p* value of distance category^f^	< 0.0001	< 0.0001	< 0.0001	< 0.0001	< 0.0001	< 0.0001	< 0.0001
Pattern of differences between distance categories^g^	A, A, B, AB, C	A, A, B, AB, C	A, A, B, AB, C	A, A, B, AB, C	A, AB, C, BC, C	AB, A, B, AB, C	A, A, B, AB, C

^a^Participants indicating that they received personal benefits were removed from the analysis

^b^The minimum aggregate annoyance value is 0 when respondents indicate either “Do not hear/see/perceive” or “Not at all annoyed” to each of the five wind turbine features

^c^The aggregate annoyance value can reach a maximum of 20 (or 16) when respondents indicate “extremely” annoyed to each of the five (or 4) wind turbine features

^d^Variance explained by the first PCA construct (max = 5, unless if only 4 variables are used then the max = 4)

^e^Distance groups in km are defined as follows: ≤ 0.550, (0.550, 1], (1, 2], (2, 5], > 5. In the analysis where distance was applied as the exposure group, the interaction between province and distance was not significant, indicating that the relationship between annoyance and distance was similar in both provinces. Nevertheless, the two provinces were analyzed separately for ease of interpretation of results

^f^*p* value based on ANOVA of the first PCA construct, assessing the relationship between the mean of construct 1 in the different distance groups

^g^Letters correspond to the distance groups (i.e., the first letter represents distance group ≤ 0.550 km, the second letter corresponds to (0.550, 1] km, etc.). Groups with the same letter are statistically similar, whereas groups with different letters are statistically different

Cronbach’s alpha was 0.82 when all 5 self-reported annoyance features were considered in an overall annoyance component. Table 3 shows the change in Cronbach’s alpha when each annoyance variable was removed from the PCA. A summary of the aggregate annoyance (after adding the scores for each self-reported annoyance) is also included in Table 3.

Based on the PCA, only the first construct had an eigenvalue greater than 1; all other constructs had eigenvalues less than 1 (data not shown). Therefore, only the first construct is considered in further analysis. When all 5 self-reported annoyance variables were considered in the analysis, the first principal component accounted for 58% of the total variance in annoyance. In PCA, *total variance* in the data set is simply the sum of the variances of these observed variables. Because they have been standardized to have a variance of 1, each observed variable contributes one unit of variance to the total variance in the data set. The relative contribution of each of the 5 annoyance features to the first construct in the PCA was as follows: blinking lights (22%), visual annoyance (22%), shadow flicker (22%), noise (22%), and vibrations (12%). Removing those who received benefits from the first construct of the PCA did not have an impact on the total variance of annoyance (i.e., it remained at 58%); however, removing each annoyance variable (individually) did alter the proportion of the variance accounted for to between 0.58 and 0.69 (Table 3). The first construct was used in an ANOVA model to determine if there was a difference in overall annoyance between the different distance groups.

### Relationship between distance and aggregate annoyance

**Fig. 1:**
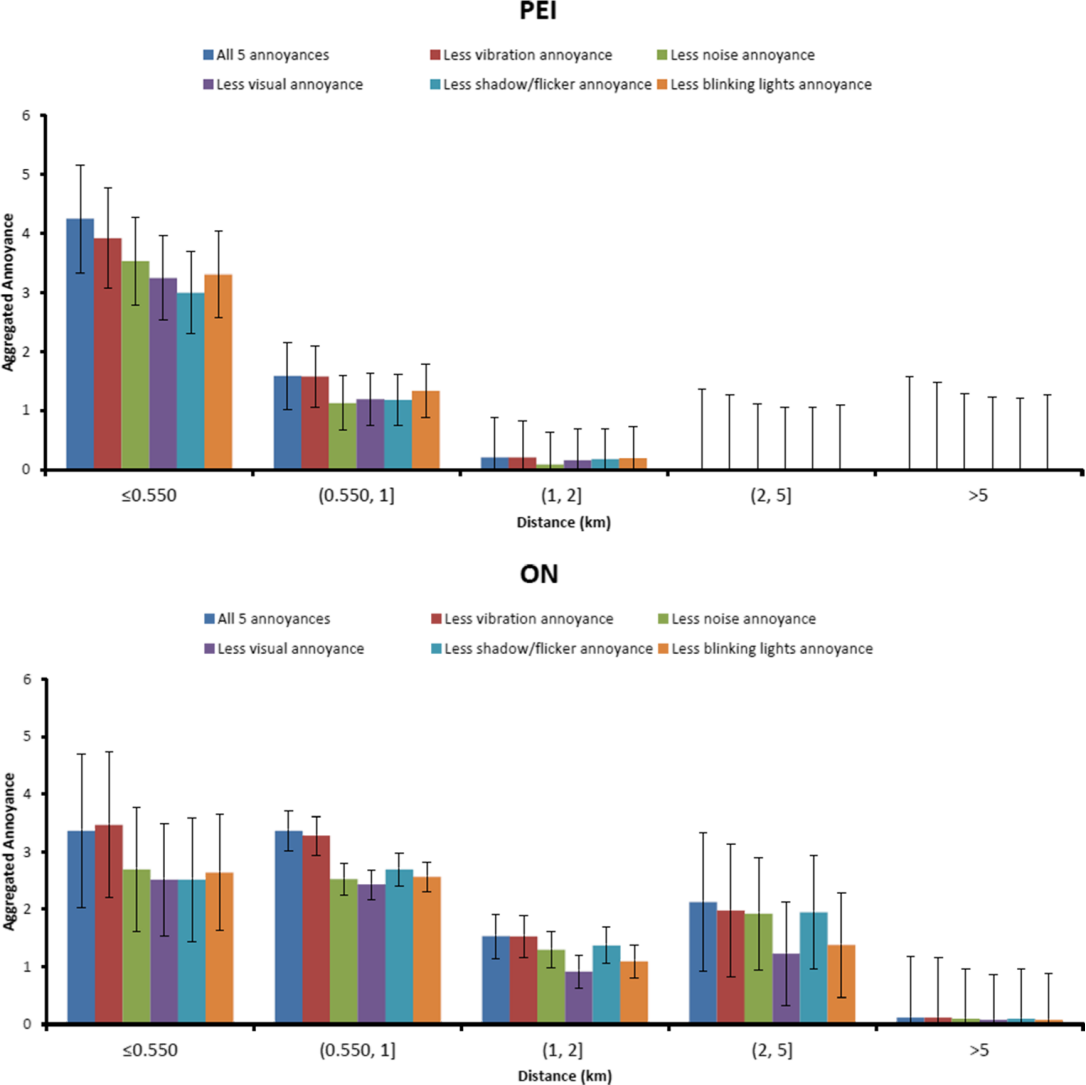
The figure illustrates the average aggregate annoyance and corresponding 95% confidence intervals based on self-reported annoyance while at home over the last year toward multiple wind turbine features. At home refers to either inside or outside the dwelling. The upper and lower panels illustrate results for PEI and ON, respectively. The full PCA is shown by the left-most bar at each exposure category (shown in blue online). The effect that removing other annoyance variables one by one had on aggregate annoyance is shown at each exposure category. The effect of removing vibration annoyance is shown in the second left-most bar (red online); the third bar from the left (green online) depicts the effect of removing noise annoyance; fourth from the left (purple online) depicts the effect of removing visual annoyance; fifth from the left (blue online) represents the effect of removing shadow flicker annoyance, and the right-most bar (orange online) shows the effect of removing annoyance toward blinking lights. The relative contribution of any given annoyance variable is reflected by the degree to which the 5-factor aggregate annoyance level drops with the removal of each annoyance variable. The larger the drop, the greater the impact the removed annoyance variable had on aggregate annoyance at that particular exposure category. Data presented also include participants reporting to receive personal benefits from having wind turbines in the area (*n* = 110) since removing these participants from the analysis did not impact the results

Figure 1 displays the exposure response relationship between aggregate annoyance (mean, 95% CI) as a function of proximity to the nearest wind turbine. In Fig. 1, aggregate annoyance is calculated as the mean of the total annoyance scores for each individual used to derive the first construct of the PCA. Upper and lower panels depict observations with all 5 annoyance variables, as a function of distance in PEI and ON, respectively. Also shown in Fig. 1 is the aggregate annoyance score after each annoyance variable has been removed, reducing the construct from 5 variables to 4 in the PCA. In all cases, there was a statistically significant increase in aggregated annoyance with decreasing distance between dwelling and turbine. Results differed slightly between ON and PEI. In the current results, confidence limits that were less than 0 were set to 0 for ease of interpretation. With all annoyance variables included, in the PEI sample, aggregate annoyance was of a similar magnitude in areas beyond 1 km, i.e., (1–2] km (mean 0.21; 95% CI 0.00, 0.88); (2–5] km (mean 0.00; 95% CI 0.00, 1.37); > 5 km (mean 0.00; 95% CI 0.00, 1.58). Annoyance significantly increased in areas between (0.550 and 1] km (mean 1.59; 95% CI 1.02, 2.15) and continued to increase further within 550 m (mean 4.25; 95% CI 3.34, 5.16). After adjustments for multiple pairwise comparisons, the adjusted *p* values indicated that there was no statistically significant difference in annoyance between (0.550 and 1] km and within 0.550 km (*p* = 0.0867). Small sample sizes in these areas (i.e., 37 participants within 0.550 km and 95 between (0.550 and 1] km) reduced the statistical power needed to detect a difference, if one existed.

In the ON sample, when compared to areas beyond 5 km (mean 0.12; 95% CI 0.00, 1.19), aggregated annoyance was significantly higher in areas between (2 and 5] km (mean 2.13; 95% CI 0.92, 3.33) with no further significant increase observed until dwellings were within (0.550–1] km of wind turbines (mean 3.37; 95% CI 3.02, 3.72). At the nearest distance category (i.e., ≤ 0.550 km), there was no additional significant increase in aggregate annoyance where the mean was 3.36 (95% CI 2.03, 4.69).

At the closest distance group (≤ 0.550 km) in PEI and ON, eliminating the annoyances one by one all had a similar effect on reducing aggregate annoyance, the exception being annoyance to vibrations, which did not result in any apparent change in aggregate annoyance. Between (0.550 and 1] km, all annoyance variables (except vibration annoyance) contributed equally to aggregate annoyance in both ON and PEI, as can be seen by the consistent drop in mean aggregate annoyance when each was removed separately. At further distances in PEI, no individual annoyance variable appeared to have a dominant impact on aggregate annoyance. In the ON sample, there was a clear reduction in aggregate annoyance between (1 and 2] km and between (2 and 5] km when visual and blinking lights annoyance was removed from the PCA. This is evidence that these two annoyances carried a large weight on total annoyance when retained in the PCA annoyance construct.

## Discussion

The conventional approach to wind turbine annoyance assessment offers a partial assessment of community response insofar as predictions focus on *high* annoyance for a single wind turbine feature. The emphasis has largely been based on noise annoyance for reasons that range from jurisdictional mandate to the implicit assumption that noise is the primary concern among a community within the vicinity of a wind turbine facility. The current analysis, which is based on a study with a large sample size and a high participation rate, accounts for a wider range of annoyance toward multiple wind turbine features. This results in an exposure response that reflects an aggregate annoyance reaction, one supported by PCA. The aggregate annoyance construct was not developed based on an assumption of equivalence between the various wind turbine features and their associated annoyance. In fact, the PCA determined how much weight each annoyance feature contributed to the aggregate annoyance construct. In the CNHS, four of the five features (noise, shadow flicker, blinking lights, and visual impacts) all loaded equally on the construct supporting the aggregate construct over any one annoyance variable in isolation. However, personal and situational factors across studies will impact this loading to the extent that these factors may change the annoyance profiles from those observed in the CNHS.

The appeal of an analysis based on proximity needs to be balanced against the fact that *distance to the nearest turbine* does not consider the wind turbine characteristics, number of turbines impacting a dwelling, or other factors that may combine to impact one’s auditory/visual perception at the dwelling, such as tree lines, or other barriers. The authors acknowledge that an exposure response based on proximity does not fully account for conditions that may influence exposure to any given wind turbine feature. There are specific cases where a dwelling can be both close to a turbine yet outside the area exposed to shadow flicker (Voicescu et al. 2016). Likewise, even at close setbacks, one’s line of sight between dwelling and turbine can be blocked by natural and/or manufactured barriers, which would reduce annoyance that depends on visual perception. Although the high negative correlation observed between distance and calculated dBA and dBC levels (Michaud et al. 2016a) adds support to an analysis based on proximity in the current study, future studies may benefit by refining the distance variable to account for situational factors that influence exposure/perception.

At the furthest distance category assessed (i.e., > 5 km), very few participants reported wind turbines as audible or visible from their dwelling. This supports the a priori decision to view this exposure category as an unexposed “control” group in the CNHS, with annoyance at this distance considered background annoyance. When compared to this area, aggregate annoyance among participants from PEI started to statistically increase when turbines were within 1 km of dwellings. Within 1 km, the relative contribution from noise, visual impacts, blinking lights, and shadow flicker on aggregate annoyance was of a similar magnitude. On the other hand, annoyance toward vibrations had no apparent influence on aggregate annoyance at any distance.

Among the ON sample, the change in aggregate annoyance with increasing distance did not follow the same pattern when compared to that observed in PEI. In ON, aggregate annoyance remained equally elevated up to 1 km, dropped significantly thereafter, but remaining statistically elevated up to 5 km. The influence that noise annoyance had on aggregate annoyance was most apparent within 1 km; at greater distances visual features became dominant. As was the case in PEI, annoyance toward vibration had no meaningful impact on overall annoyance at any setback distance assessed. The near-absent annoyance response toward vibrations confirms perception of vibration/rattles was reported by very few participants, which is consistent with our estimates that the sound pressure level in low frequencies was below the ANSI recommended thresholds (American National Standards Institute (ANSI) 2008) for reducing moderately perceptible rattle/vibration and the annoyance that rattle/vibration may cause (Health Canada 2014).

It is worth considering the possibility that insight gained from an analysis based on high WTN annoyance may be lost if annoyance is reported as a change in aggregate annoyance. When comparisons between the participants drawn from PEI and ON are limited to *high* WTN annoyance, the prevalence of annoyance within 550 m of a turbine was higher in PEI (13.9% (95% CI (6.1, 28.7)) when compared to ON (5.9% (95% CI (1.6, 19.1)). With aggregate annoyance as the outcome, annoyance remains higher in PEI; however, the provincial samples differ by a lesser magnitude (i.e., aggregate annoyance in PEI 4.25 (95% CI (3.34, 5.16)), compared to 3.36 (95% CI (2.03, 4.69)) in ON). Which of the two approaches provides the “correct” account of the provincial difference in wind turbine annoyance is not subject to a statistical test, but it is of interest that they appear more similar when comparisons are based on responses that go beyond high noise annoyance. Therefore, in the current study, an analysis based on aggregate annoyance retains important information in the pattern of change between the two provinces with respect to wind turbine exposure, while still accounting for total annoyance toward a wide range of wind turbine features.

Assigning relevance to the observed change in aggregate annoyance at any given setback distance is difficult for two reasons. First, the observed changes in aggregate annoyance do not correspond to previously reported changes in *high* annoyance and the current analysis makes no attempt to define an aggregated annoyance value that would be equivalent to *high* annoyance. Second, although aggregate annoyance remained significantly elevated above background annoyance up to 1 km in PEI and up to 5 km in ON, the absolute magnitude of annoyance needs to be considered to provide perspective. Where 20 is the maximum aggregate annoyance score, the highest average level observed in PEI and ON was 4.25 and 3.36, respectively. However, it should be underscored that a value of 20 corresponds to the highest magnitude of annoyance on each of the 5 annoyance features, where *extreme* annoyance was assigned a value of 4.

As this area of research matures, new findings may identify an aggregate annoyance value that corresponds to a threshold for community acceptability. Part of the widespread adoption of *high noise annoyance* as a targeted outcome for community noise in general is that the burden of disease associated with it has been quantified by the World Health Organization (WHO) (2011). High noise annoyance has repeatedly been shown to have a statistical association with long-term average sound levels and other health measures (Michaud et al. 2016a; Niemann et al. 2006). As data in this area accumulate, there is no reason why a similar approach could not eventually be adopted for aggregate annoyance or a change in the aggregate annoyance score. A mean aggregate annoyance score that could reliably distinguish participants who report additional health effects (or noise complaints) from those who do not could be one of several factors considered by jurisdictions responsible for decisions regarding wind turbine developments. An assessment of aggregate annoyance in this context has been presented by the authors in an accompanying publication (Michaud et al. 2018).

A possible application for aggregate annoyance would be where there is an interest in tracking and reporting total annoyance over time. Situations where such an approach might be considered could include various stages of project development and/or where there is a need to estimate the impact of mitigation measures. As shown here, one can isolate the relative contribution that any given wind turbine feature has on total annoyance, which could identify where mitigation resources are best allocated. As discussed above, there may also be value in assessing aggregate annoyance when two communities initially appear to differ by a large margin in their annoyance response toward a single wind turbine feature, and where the comparison has been limited to high annoyance.

Despite the promise that may be afforded by an all-encompassing annoyance metric in terms of quantifying overall community annoyance toward wind turbines broadly, this is the first analysis of this nature and it would be premature to over-interpret the current findings. Until the science base in this area matures, the best application for aggregate annoyance may be as a supplement to traditional analyses that are based on high annoyance.

## Electronic supplementary material

ESM 1 (DOCX 164 kb)
