# Supplementary material for: Derivation and application of a composite annoyance reaction construct based on multiple wind turbine features
Source: Can J Public Health. 2018 Apr 11;109(2):242–51. doi: 10.17269/s41997-018-0040-y (PMC6019414; doi:10.17269/s41997-018-0040-y)
Supplement: Supplementary file 1 — (DOCX 164 kb) [file 41997_2018_40_MOESM1_ESM.docx]

**Background**

The following supplemental material provides the principal component analysis (PCA) results as a function of calculated outdoor A- and C-weighted wind turbine noise (WTN) levels. The analysis is provided for comparative purposes only. The authors consider an exposure parameter that is based on proximity to wind turbines as more appropriate when 4 of the 5 annoyance variables are non-acoustical in nature. It is nevertheless acknowledged that a calculated and/or measured noise metric will always be more appropriate when *noise* *annoyance* is the sole variable of interest because parameters that influence sound pressure levels at the dwelling will be more precisely accounted for (e.g., topography, type of turbine, number of turbines) with a noise metric.

**Results**

Table 1 presents summary statistics of aggregated annoyance (sum of annoyance scores based on the annoyances used to derive the construct in PCA). Analysis of variance (ANOVA) models were used to compare the first construct of PCA (based on the various variables of annoyance) in the different WTN level categories (either A or C-weighted). Unfortunately, the range of calculated C-weighted values was not wide enough to permit more than 3 WTN level categories.

**Supplemental Table 1. Full and reduced models of aggregate annoyance**

|  | **Variable removed from full PCA** | | | | | | |
| --- | --- | --- | --- | --- | --- | --- | --- |
|  | None  (full model) | Personal benefits^1^ | Vibration annoyance | Noise annoyance | visual annoyance | shadow/flicker annoyance | blinking lights annoyance |
| Cronbach's alpha | 0.82 | 0.82 | 0.85 | 0.76 | 0.75 | 0.76 | 0.75 |
| Summary statistics for factor based on the addition of annoyance variables | | | | | | | |
| N | 1226 | 1116 | 1233 | 1226 | 1226 | 1227 | 1226 |
| Mean | 2.25 | 2.36 | 2.20 | 1.75 | 1.56 | 1.83 | 1.69 |
| Median | 0.00 | 0.00 | 0.00 | 0.00 | 0.00 | 0.00 | 0.00 |
| Std Dev | 3.89 | 3.98 | 3.75 | 3.14 | 2.90 | 3.13 | 2.97 |
| Std Error | 0.11 | 0.12 | 0.11 | 0.09 | 0.08 | 0.09 | 0.08 |
| Minimum^2^ | 0 | 0 | 0 | 0 | 0 | 0 | 0 |
| Maximum^3^ | 20 | 20 | 16 | 16 | 16 | 16 | 16 |
| Values from PCA |  |  |  |  |  |  |  |
| Eigenvalue of construct 1^4^ | 2.91 | 2.91 | 2.77 | 2.35 | 2.32 | 2.36 | 2.31 |
| Proportion explained by construct 1 | 0.58 | 0.58 | 0.69 | 0.59 | 0.58 | 0.59 | 0.58 |
|  | **PEI dBA^5^** | | | | | | |
| p-value of WTN categories^6^ | <0.0001 | <0.0001 | <0.0001 | <0.0001 | <0.0001 | <0.0001 | <0.0001 |
| pattern of differences between WTN categories^7^ | A, AB, A, BC, C | A, AB, A, BC, C | A, AB, A, BC, C | A, AB, A, BC, C | A, AB, A, B, B | A, AB, A, B, B | A, AB, A, B, B |
|  | **ON dBA^5^** | | | | | | |
| p-value of WTN categories^6^ | <0.0001 | <0.0001 | <0.0001 | <0.0001 | <0.0001 | <0.0001 | <0.0001 |
| pattern of differences between WTN categories^7^ | A, A, A, B, B | A, A, A, B, C | A, A, A, B, B | A, AB, A, BC, C | A, A, A, B, C | A, A, A, B, B | A, A, A, B, B |
|  | **PEI dBC^8^** | | | | | | |
| p-value of WTN category^6^ | <0.0001 | <0.0001 | <0.0001 | <0.0001 | <0.0001 | <0.0001 | <0.0001 |
| pattern of differences between WTN categories^9^ | A, A, B | A, A, B | A, A, B | A, A, B | A, A, B | A, A, B | A, A, B |
|  | **ON dBC^8^** | | | | | | |
| p-value of WTN category^6^ | <0.0001 | <0.0001 | <0.0001 | <0.0001 | <0.0001 | <0.0001 | <0.0001 |
| pattern of differences between WTN categories^9^ | A, B, C | A, B, C | A, B, C | A, B, C | A, A, B | A, B, C | A, B, C |

^1^Participants indicating that they received personal benefits were removed from the analysis; ^2^The minimum aggregate annoyance value is 0 when respondents indicate “not at all annoyed” to each of the 5 wind turbine features; ^3^The aggregate annoyance value can reach a maximum of 20 (or 16) when respondents indicate “extremely” annoyed to each of the 5 (or 4) wind turbine features. ^4^Variance explained by the first PCA construct (max = 5, unless only 4 variables are used then the max=4); ^5^dBA WTN categories are defined as follows: <25, [25 – 30), [30 – 35), [35 – 40), ≥40. ^6^ P-value based on ANOVA of the first PCA construct, assessing the relationship between the mean of construct 1 in the different WTN groups. ^7^ Letters correspond to the WTN groups (i.e., the first letter represents WTN group <25 dBA, the second letter corresponds to [25 – 30) dBA, etc.). Groups with the same letter are statistically similar, whereas groups with different letters are statistically different; ^8^WTN level categories in dBC are defined as follows: <50, [50 - 55), ≥55. ^9^Letters correspond to the WTN levels (i.e., the first letter represents WTN level <50dBC, the second [50-55) dBC, the third ≥55 dBC). Groups with the same letter are statistically similar, whereas groups with different letters are statistically different.

|  |
| --- |
| In all cases (whether 4 or 5 self-reported annoyance variables are used, with or without those who receive benefits), the lowest aggregated annoyance scores were observed in areas where the A-weighted WTN levels were below 25 dB and where C-weighted WTN levels were below 50 dB. As shown in Supplemental Material Figure 1, the overall annoyance score increased statistically with increasing A- or C-weighted WTN levels. For the A-weighted WTN levels, annoyance scores were statistically similar across the three categories spanning <25-35 dB. In areas where A-weighted WTN levels were the highest [40-46] dBA, the average overall annoyance (where 20 was the maximum score with all 5 annoyance variables) was 2.87 (95% CI: 2.15, 3.59) in PEI and 3.83 (95% CI: 3.24, 4.42) in ON. In areas where the C-weighted WTN levels exceeded 55 dB, the average overall annoyance with all 5 annoyance variables included (where the maximum score would be 20) was 2.19 (95% CI: 1.70, 2.68) in PEI and 3.24 (95% CI: 2.92, 3.55) in ON.  **Supplemental Figure 1. Aggregate annoyance by calculated outdoor wind turbine noise level**  a.  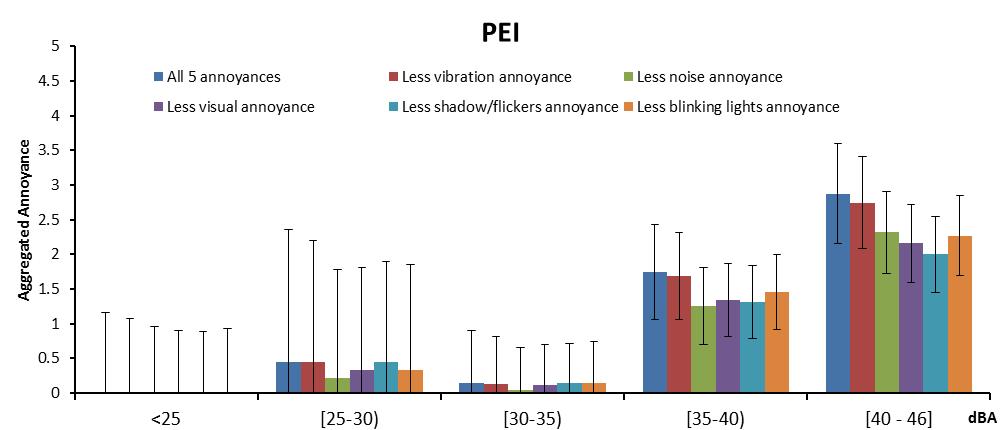  b.  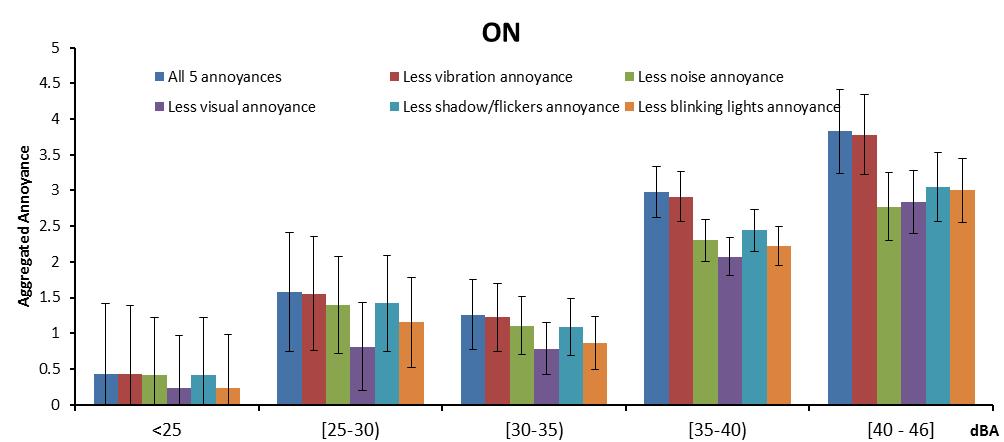  c.  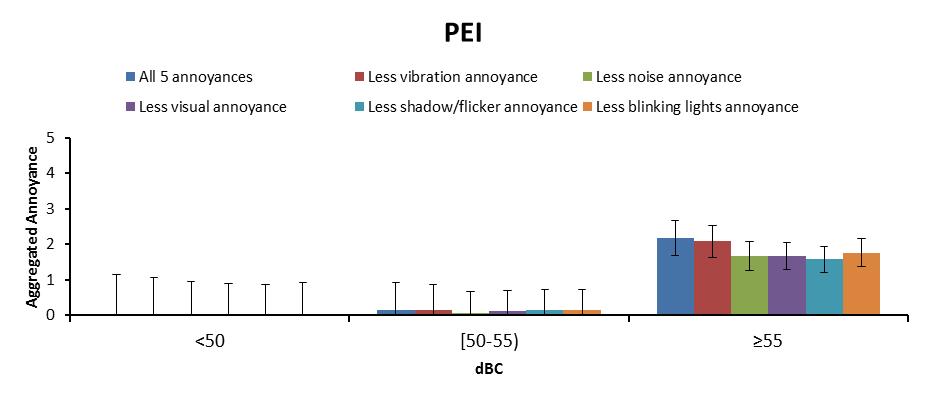  d.  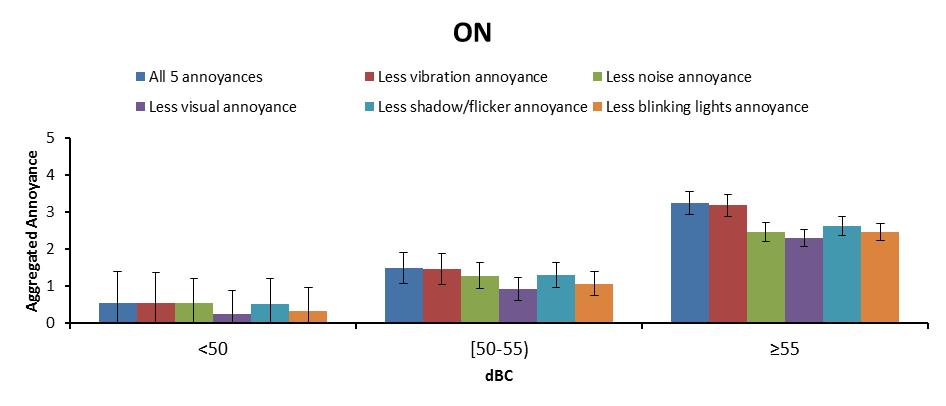 |
|  |

The figure illustrates the average aggregate annoyance and corresponding 95% confidence intervals based on self-reported annoyance while at home over the last year toward multiple wind turbine features, by province. At home refers to either inside or outside the dwelling. Panels a and b illustrate results for noise, visual impacts, blinking lights, shadows or flickers and vibrations or rattles perceived indoors during wind turbine operations as a function of calculated outdoor A-weighted wind turbine sound levels in PEI and ON, respectively. Panels c and d illustrate results for noise, visual impacts, blinking lights, shadows or flickers and vibrations or rattles perceived indoors during wind turbine operations as a function of calculated outdoor C-weighted wind turbine sound levels in PEI and ON, respectively. Each panel presents the full PCA with all 5 annoyance variables retained in the analysis. The effect that removing other annoyance variables one by one had on aggregate annoyance is shown at each exposure category. The effect of removing vibration annoyance is shown in the 2^nd^ leftmost bar (red online); the 3^rd^ bar from the left (green online) depicts the effect of removing noise annoyance; 4^th^ from left (purple online) depicts the effect of removing visual annoyance; 5^th^ from left (turquoise online) represents the effect of removing shadow flicker annoyance and the rightmost bar (orange online) shows the effect of removing annoyance toward blinking lights. The relative contribution of any given annoyance variable is reflected by the degree to which the 5 factor aggregate annoyance level drops with the removal of each annoyance variable. The larger the drop, the greater the impact the removed annoyance variable had on aggregate annoyance at that particular exposure category. Data presented also includes participants reporting to receive personal benefits from having wind turbines in the area (n=110) since removing these participants from the analysis did not impact the results.
